# COVID-19 Quarantine Impact on Wellbeing and Cognitive Functioning During a 10-Week High-Intensity Functional Training Program in Young University Students

**DOI:** 10.3389/fnbeh.2022.822199

**Published:** 2022-04-08

**Authors:** Juan Arturo Ballester-Ferrer, Laura Carbonell-Hernández, Diego Pastor, Eduardo Cervelló

**Affiliations:** Sports Research Centre, Department of Sport Sciences, Miguel Hernández University of Elche, Elche, Spain

**Keywords:** reaction-time, exercise, COVID-19, wellbeing, HIFT, quarantine

## Abstract

Physical exercise can improve cognitive functioning and wellbeing; however, the degree of change in either of these two variables seems to be related to the exercise intensity or type. Therefore, new physical training (PT) programs have been developed to increase exercise efficiency. One such example is high-intensity functional training (HIFT), which has proven to be a time-efficient and highly effective strategy to improve physical fitness. This study analyzed whether HIFT can affect reaction time (RT) and vitality, as well as positive and negative affect. Forty-two college students participated in the study, 21 in the experimental group and 21 in the control group. The experimental group completed 10 weeks of training, five of which were supervised, and the remainder consisted of online training during the COVID-19 quarantine. Participants were evaluated at the beginning, at the end of the 5 weeks of supervised training, and after the 5 weeks of online training. HIFT improved RT without changes in psychological wellbeing during the entire period of training supervised and online. Therefore, during the HIFT program, the quarantine situation did not adversely affect this population’s wellbeing, but it did negatively affect adherence to the training program.

## Introduction

It is well-known that acute and chronic physical exercise (PE) affects physical and physiological functions and influences aspects related to cognitive functioning. According to Herold et al. (2019), it is crucial to distinguish between physical activity (PA), acute and chronic PE, and physical training (PT). All muscle activity that increases energy expenditure is PA (Herold et al., 2019). PE is a specific, planned and structured PA. PE may include a single bout (acute PE) or repeated bouts over a short-term or long-term period (chronic PE). Finally, when chronic PE is purposed to increase (or maintain) one or multiple dimensions of fitness, this is known as PT (Herold et al., 2019).

Regarding exercise and cognitive functioning, PA and fitness have been linked to the academic performance of young students (Marques et al., 2018), inhibitory function in young and old subjects (Peruyero et al., 2017; Carbonell-Hernández et al., 2019; Pastor et al., 2019), memory (Erickson et al., 2011; Jeon and Ha, 2017), wellbeing (Cervelló et al., 2014), depression (McMahon et al., 2017), and other cognitive outputs. However, it is not well-known how different variables of the exercise session (e.g., exercise intensity, exercise duration, exercise type) can modulate it. The literature on exercise and cognition has traditionally dealt with non-modifiable parameters like sex or genotype. Less research is available on the modifiable parameters, like the individual dose-response relationship (Herold et al., 2019).

Regarding the dose-response relationship of exercise to improve cognitive functioning and wellbeing, it is clear that exercise intensity does matter. According to wellbeing, low-intensity but not high-intensity aerobic exercise seems more effective in producing acute changes in wellbeing in adolescents (Pastor et al., 2019). However, high-intensity chronic exercise in university students is more efficient in reducing stress and improving wellbeing (Leuchter et al., 2022).

Regarding cognitive functioning, high-intensity aerobic exercise seems to produce greater improvements in other age groups and different cognitive domains like choice reaction time (RT) (Chang et al., 2012). Furthermore, in favor of high-intensity acute exercise, it has been shown that high intensity can produce greater increases in neurotrophins like brain-derived neurotrophic factor (BDNF) (Saucedo Marquez et al., 2015). BDNF promotes neurogenesis, synaptic plasticity, dendritic and axonal growth, and cell survival (Walsh et al., 2020). The BDNF increase with exercise intensity may be related to lactate release (Ferris et al., 2007). It is well-known that high-intensity exercise performance produces a considerable release of lactate (Ferguson et al., 2018), and high-intensity functional training (HIFT) implies high-intensity exercise. Moreover, lactate release is also related to cerebral blood flow and improves neurogenesis, neuroprotection, neuronal plasticity, and memory (Hashimoto et al., 2021).

Moreover, resistance training can also improve cognitive functioning (Landrigan et al., 2020); single bouts of resistance exercise showed more significant improvements when higher loads were used (Chang and Etnier, 2009). However, the optimal exercise and training characteristics to effectively enhance cognitive functioning with chronic PE programs are relatively unknown (Soga et al., 2018). So, it seems that a single bout of both high-intensity aerobic or resistance exercise can acutely improve RT. However, we do not know if high-intensity exercise would also be effective when applied in chronic PE programs. It is possible that continuous exposure to lactate, in turn, could induce the release of BDNF to produce such an effect (Hashimoto et al., 2021).

Regarding changes in cognitive functioning, PT and acute PE have been shown to improve attention processes, information processing speed, and executive control (Colcombe and Kramer, 2003; Tsai et al., 2014; Pontifex et al., 2015). Among the various tools generally used to determine the impact of PE on the above-mentioned cognitive variables, RT has been used in the literature to evaluate cognitive and motor functions, as it involves both central and peripheral components (Ozyemisci-Taskiran et al., 2008). RT is understood as the time a person needs to initiate a movement (Magill, 2010). There are different types of RT. On the one hand, simple RT occurs when only one possible stimulus requires a single type of response (Posner and McLeod, 1982), whereas in more complex choice RT, there are different possibilities to choose from during the experiment, as it involves the appearance of different stimuli that require different responses (Neubauer, 1990). More specifically, RT measures processing speed (Mulder and van Maanen, 2013), and this variable reflects a person’s cognitive functioning (Deary et al., 2010). At the morphological level, processing speed may be an indirect measure of white brain substance, as changes in this substance are associated with changes in processing speed (Gunning-Dixon et al., 2009). At the behavioral level, it has recently been shown that medical students with better academic performance had lower RT (Prabhavathi et al., 2017). Not only that, in middle-aged and older people, lower performance in RT tasks can be a predictor of heart disease and stroke mortality (Shimizu et al., 2018).

Like in cognition, PA can improve wellbeing in young students (McMahon et al., 2017). Nowadays, wellbeing is a European priority (Miret et al., 2015), but there is no clear consensus about its definition or operationalization (Korhonen et al., 2014). Positive or negative indicators such as self-esteem, quality of life, positive and negative affect or vitality are usually used (Pastor et al., 2019). Some authors consider two perspectives for studying wellbeing: the hedonic perspective, which considers wellbeing as the presence of positive affect and the absence of negative affect; and the eudaimonic perspective, which associates wellbeing with the possibility of performing or expressing the most valuable human potentials, related to optimal psychological functioning (Ryan et al., 2013). Moreover, in both young and older individuals, it has been seen that cognitive performance changes could be affected by aspects such as mood and social relations (Lieberman et al., 2014). In addition, cardiorespiratory fitness (CRF) is related to vitality in young people (Eddolls et al., 2018), and vitality should correlate with positive and negative affect (Cervelló et al., 2014). Thus, the improvement of CRF with exercise training could improve wellbeing.

Consequently, it would be interesting to determine how PE can affect wellbeing and processing speed. At the acute level, improvements have been reported in complex RTs after sub-maximum exercise in university students (Audiffren et al., 2008; Ashnagar et al., 2015) and in adolescents’ wellbeing (Pastor et al., 2019). On the other hand, some studies have observed a relationship between fitness and RT in young students (Luque-Casado et al., 2013; Wang et al., 2015; Maghsoudipour et al., 2018), although others did not find this association (Moradi et al., 2019). At the chronic level, the effects of the exercise intensity, duration, and frequency to produce long-term effects on processing speed and wellbeing in this population are not well-established.

Regarding exercise programs, the need to investigate new training methods to improve several fitness components simultaneously and efficiently, without high volumes of training and favoring adherence, has been indicated previously (Heinrich et al., 2014). In this sense, HIFT has recently emerged. HIFT is defined as a training style that incorporates various functional movements performed at high exercise intensity and is designed to improve general physical fitness and performance parameters (Feito et al., 2018). Unlike HIIT, this type of training combines aerobic activities with exercises to improve muscle strength (Feito et al., 2018). Among its benefits are improved body composition, power, muscle strength, and aerobic capacity, the same as or even more so than when performing continuous workouts or HIIT (Feito et al., 2018). However, the term “functional” may not be correct in this training style (Ide et al., 2021), as HIFT can be more clearly described as combined or concurrent training. Therefore, like a high-intensity aerobic and resistance-training regime, it seems a powerful candidate to improve RT and wellbeing in young people. Moreover, high-intensity exercise can release high amounts of lactate, a molecule positively related to cognitive function (Hashimoto et al., 2021).

Therefore, this study’s main objective was to determine whether a supervised HIFT group program of 10 weeks could improve processing speed and wellbeing. However, as a consequence of the COVID-19 pandemic, a national lockdown was announced in the middle of the program (week five), and supervised training had to be terminated. Thus, online training was implemented for the next 5 weeks.

The COVID-19 pandemic impacted the economy and society in many aspects, disturbing lifestyle and health behaviors (Dragun et al., 2021). The lockdown deteriorated daily activities, especially physical activities (Dragun et al., 2021). In addition, as a consequence of the quarantine, there was a negative impact on the population’s wellbeing (Brooks et al., 2020), with higher levels of anxiety and depression (Burke et al., 2020).

The study’s second objective arose as a consequence of the COVID-19 quarantine. We aimed to determine whether the “stay-at-home” orders and the need to continue training at home individually, adapting it to the new situation, would impact cognitive functioning and wellbeing in the participants.

Thus, we hypothesized that the 5-week HIFT supervised training phase would improve participants’ RT and psychological wellbeing and give rise to high adherence rates. Conversely, the phase carried out during the 5 weeks of strict quarantine due to COVID-19 was expected to decrease wellbeing compared to the in-person, supervised program, coupled with lower adherence and a reduction in RT improvement.

## Materials and Methods

### Participants

The sample consisted of 42 college students, 21 in the experimental group (14 males and 7 females) and 21 in the control group (9 males and 12 females). All the participants were adults over 18 years old, and all of them claimed to be sedentary (one or fewer days of PE a week) during at least the last 6 months. In addition, all participants completed an initial questionnaire to ensure they met the requirements to be part of the study, such as not having recently suffered injuries and not being involved in any other training program. Their sociodemographic characteristics are summarized in Table 1.

**TABLE 1:** Anthropometric and fitness characteristics of the participants (*N* = 42).

Characteristics	Experimental (*n* = 21)	Control (*n* = 21)
Age (years)	19.71 ± 1.71	21.52 ± 2.74
Weight (kg)	69.17 ± 8.04	66.57 ± 13.57
Height (m)	1.72 ± 0.09	1.69 ± 0.08
BMI (kg/m^2^)	23.20 ± 2.25	23.20 ± 3.73
VO_2_ max (ml kg^–1^min^–1^)	43.50 ± 5.42	
MAS (km/h)	14.20 ± 1.75	15.47 ± 2.91
HR max (beats min^–1^)	200.90 ± 7.99	193.60 ± 8.16

*All values are expressed as mean ± SD. BMI, body mass index; VO_2_ max, maximum oxygen consumption; HR max, maximum heart rate; MAS, maximal aerobic speed.*

### Measurements

#### Cardiorespiratory Function: Stress Test

Participants of the experimental group performed a maximum incremental test on a treadmill with a measure of oxygen consumption (VO_2_) and heart rate (HR) throughout the test to determine the maximum VO_2_ (VO_2_ max), maximum HR (HR max), and their associated values in the first (VT1) and second ventilatory threshold (VT2). The exchange of respiratory gases was measured using the MasterScreen CPX analyzer (Hoechberg, Germany) breath by breath after being calibrated. The VO_2_ max was calculated as the highest 30-s mean of VO_2_. In addition, 15-s means of O_2_ and CO_2_ were used to determine VT1 and VT2 (Pettitt et al., 2013). Participants were not allowed to drink or speak during the test and were asked to refrain from intense exercise 24 h earlier.

The incremental test protocol consisted of a 3 min warm-up at 5 km/h with a 1% slope. Then, the test was performed on a 1% slope and began at a speed of 6 km/h and increased 1 km/h per minute until fatigue.

The incremental test protocol could not be repeated at the end of the study due to the COVID-19 lockdown, so no data regarding the possible fitness improvements of the participants was obtained.

#### Cognitive Functioning

##### Reaction Time

A test of increasing difficulty was used to measure the time required to respond to a stimulus. All the participants were requested to complete the test in the afternoon at home. The test was carried on a day with no exercise training session in a quiet place. A digital application with three tests was used in the study (AppAndAbout, 2019). Each test consisted of passing ten trials of different stimuli, and between each trial, there was a random rest period between 1,000 and 1,500 ms. The first test, the Training Test (TrT), was used to train the participants at the beginning of each measure. In the TrT, a green or red luminous stimulus was presented, and the subject had to press a green button only if the stimulus presented was of the same color. In the second test, the Choice Reaction Test (CRT) (ICC = 0.78, SEM = 0.05 s), there were also two luminous signals, red and green, and two buttons, red and green. The subject had to press the button with the matching color corresponding to the luminous signal presented (e.g., if the light signal was red, they had to press the red button). The third test, the Interference Test (InT) (ICC = 0.80, SEM = 0.05 s), consisted of introducing interference in the decision process of the second test: the participants had to respond in reverse, such that the signal should not coincide with the color of the button (e.g., if the light signal was green, they must press the red button). The third test was the most complex because it added interference. A familiarization protocol was used at the beginning of the study, repeating all tests five times. The familiarization protocols of the first 15 participants were analyzed to calculate the ICC of the tests, with moderate to strong reliability (Wells et al., 2014).

#### Psychological Wellbeing

##### Subjective Vitality

The Subjective Vitality Questionnaire (Ryan and Frederick, 1997) was used to measure the perception of vitality before and after the two phases of the training program, adapted to Spanish by Molina-García et al. (2007). This questionnaire can be considered a measure of psychological wellbeing (Ryan et al., 2013). This instrument comprises seven items that indicate how one feels at present (e.g., I feel alive and vital). The responses are rated on an eight-point Likert-type scale ranging from 0 (*not completely true*) to 7 (*very true*). Cronbach’s alpha in the different experimental situations was comprised between 0.88 and 0.95.

##### Affective State

Following each training program phase, participants were asked to complete the *Positive and Negative Affect Schedule* (Mackinnon et al., 1999), which assesses their positive and negative feelings. This questionnaire is considered a hedonic measure of wellbeing. The scale is made up of nine adjectives, which are grouped into two factors in response to the item “Indicate how you feel right now….” Four of its items are associated with *Positive Affect* (“joyful, happy, content, amused”) and five with *Negative Affect* (“depressed, worried, frustrated, angry, unhappy”). The responses are rated on an eight-point Likert-type scale ranging from 1 (*not completely*) to 7 (*extremely*). Cronbach’s alpha for Positive Affect was between 0.89 and 0.95, and for Negative Affect between 0.83 and 0.92.

### Adherence to the Training Program

Participation was monitored through a registration sheet in the supervised sessions and a mobile application during the quarantine phase. In addition, each person recorded the session and their perception of effort (RPE: rate of perceived exertion) 30 min after completing the session by the modified RPE-10 scale. A total of 30 sessions was performed, 15 in each of the phases of the program (supervised and online phases). Adherence was considered sufficient if at least 90% of the sessions were completed (Heinrich et al., 2014).

### Procedure

The physical evaluation was carried out at the beginning of the program, and RT and wellbeing evaluations were conducted before the start of the training program (January–February 2020), after 5 weeks of the supervised group intervention (March 2020), and after 5 weeks of individual training during the quarantine period due to the COVID-19 global pandemic (April 2020). The experimental procedure for this study followed the latest (7th) Helsinki Declaration and was approved by the University Ethics Committee (UMH.CID.DPC.02.17). Study participants gave their informed written consent before participating in the study and were informed of the confidentiality and anonymity of the results obtained.

**FIGURE 1:**
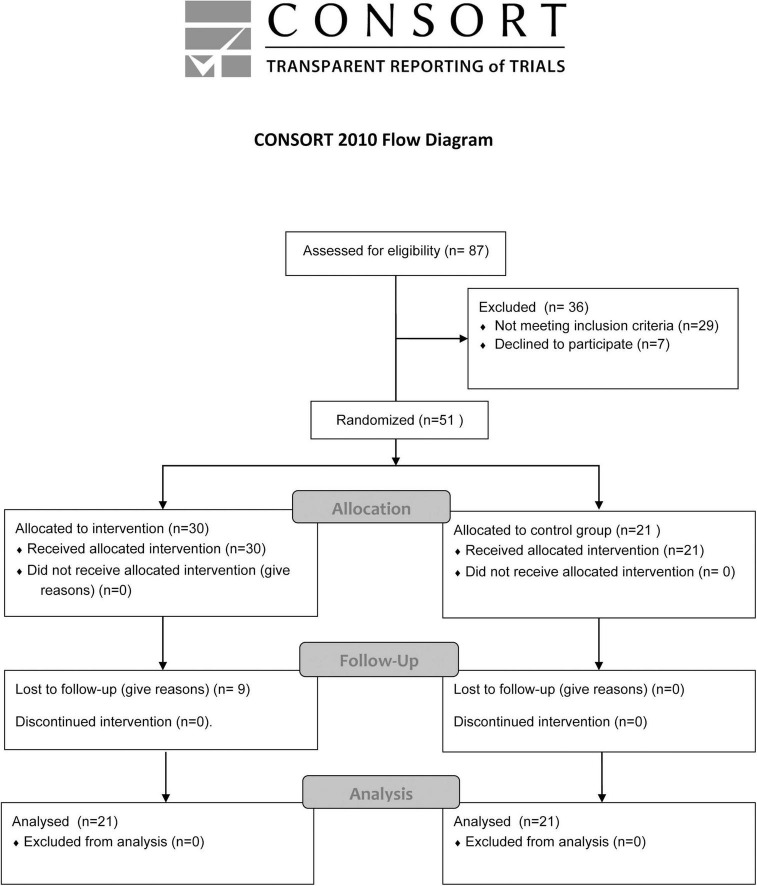
Consort flow chart.

The participants’ experimental allocation was distributed as is shown in the CONSORT flow chart (Figure 1). The inclusion criteria were (a) did not participate in any PE program simultaneously, (b) physical inactivity in the last 6 months, and (c) did not suffer injuries in the last 6 months. The participants were randomized using Microsoft Excel software with the “Random” function. There was no allocation concealment.

The sample size was calculated *post-hoc* using G-Power (G*Power software, ver. 3.1.9.7; Heinrich-Heine-Universität Düsseldorf, Düsseldorf, Germany).^1^ It was calculated for the RT analysis. For an ANOVA-RM 2 × 3, and with 42 participants, it is necessary an effect size of η_*p*_^2^ > 0.04 to ensure a statistical power (1–β) > 0.8 (α = 0.05, correlation among repetitive measures = 0.45). Results have a positive power with η_*p*_^2^ > 0.04.

#### Training Program

Two research group members prescribed the supervised training program and carried it out. The sessions were 1 h long, 3 days a week (Monday, Wednesday, and Thursday). The sessions were conducted at university facilities, dividing the participants into two different time schedules to ensure the availability of the material. The training sessions during the quarantine phase were sent to participants *via* a mobile application to be performed at home.

The sessions were divided into four parts: (a) 10 min of a standardized warm-up; (b) a strength-block of about 15 min, occasionally, on a specific movement pattern with load requirements as a function of the manifestations of muscular endurance or hypertrophy depending on the week; (c) HIFT as the primary training block, which ranged from 10 to 30 min; and (d) cool down through mobility exercises and 10 min of static stretches. Repetition methods (completing a series of exercises in the shortest possible time, or maximum repetitions in a scheduled time) or time methods (rest-work ratios similar to the traditional HIIT method) were used in the main HIFT block. This block included cyclic exercises, such as running or jumping rope, and muscle endurance exercises with bodyweight or light loads (e.g., squats, swings, med ball throws, push-ups, pull-ups, jumps, and more) (Feito et al., 2018). The exercise intensity was all-out for all, and the recovery was self-paced, self-selected, and passive (Feito et al., 2018). The training sessions were adapted to the new situation during the quarantine period. Thus, work with loads was prescribed using generic materials that anyone may have at home (bags of rice, bottles of water, milk containers, and other common materials) (Jiménez-Pavón et al., 2020). The days and hours of training were the same during the presential and quarantine periods.

#### Monitoring and Quantification of Training

The exercise intensity of the presential training sessions was rated with two different methods, one objective and one subjective: HR and subjective RPE-10 scale of the session. Only RPE was used during the sessions during quarantine.

Heart rate was registered during the sessions with the Polar Team2 Pro System (Polar Electro Oy, Kempele, Finland), which includes 20 coded chest straps, allowing HR registration every second. The recorded HR was used to calculate a load of each session, using Lucia’s Training Impulse (TRIMPs) method (Lucía et al., 1999), which uses the HR at the individual ventilatory thresholds obtained in the stress test to establish three training zones: Zone 1 “below VT1,” Zone 2 “between VT1 and VT2,” and Zone 3 “above VT2.” For the calculation of the TRIMP, the time expended in each zone is multiplicated by the zone number (1, 2, or 3).

The modified 10-point scale was used to quantify the internal load through the RPE (Foster et al., 2001). Participants were asked to determine the exercise intensity of the training 30 min after completing it.

### Statistical Analysis

Repeated-measures ANOVA was performed, followed by a *post-hoc* test with Bonferroni adjustment to detect changes between the different time measurements for CRT, InT, positive affect, negative affect, vitality, and attendance. The bilateral significance level was set at *p* < 0.05. Sphericity was evaluated with Mauchly’s sphericity test. The effect sizes are expressed as partial eta-squared (η_*p*_^2^) and are grouped as small (≤0.01), medium (≤0.06), and large (≤0.14) (Cohen, 1992). Paired *t*-test were used to analyze the exercise intensity of the sessions. A Pearson correlation was performed to analyze the relations of the wellbeing questionnaires. The results were analyzed with JASP 0.16 software (Eric-Jan Wagenmakers, Department of the Psychological Methods University of Amsterdam, Nieuwe Achtergracht 129B, Amsterdam, Netherlands).

## Results

**FIGURE 2:**
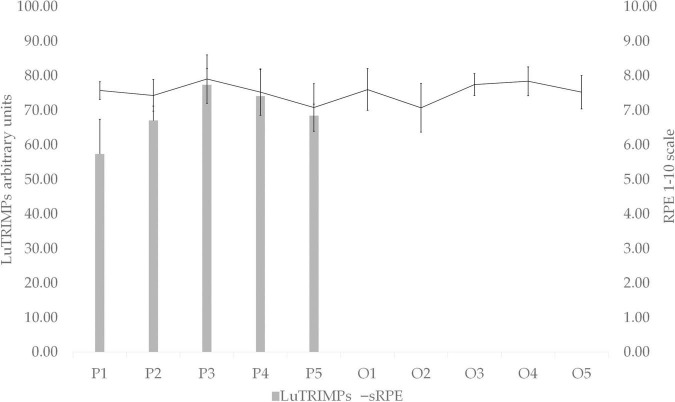
Weekly (1–5) load in presential sessions (P) and online sessions (O) during quarantine periods expressed in RPE and LuTrimp. *n* = 21.

Perception of effort was maintained with similar values during the online training sessions and the supervised training sessions (Figure 2). Moreover, during the supervised training sessions, the evolution of RPE was similar to LuTRIMP evolution (Figure 2). Despite changes in the structure of the sessions between the two phases, no significant differences in the exercise intensity of the sessions were observed through the recording of RPE between the supervised sessions and online sessions or between sessions (Figure 2), analyzed with paired *t*-tests (*p* = 0.39). Therefore, the stabilization of improvements in RT and the decrease in adherence between the two phases of the program were not attributable to load differences in the prescribed sessions, so there may be other underlying factors.

The repeated-measures ANOVA shows the interaction between TIME at the three time points [pretest (PRE), after 5 weeks of supervised training (POST 5), and after 5 weeks of online training (POST 10)] and GROUPs [experimental group (exp), control group (con)].

**FIGURE 3:**
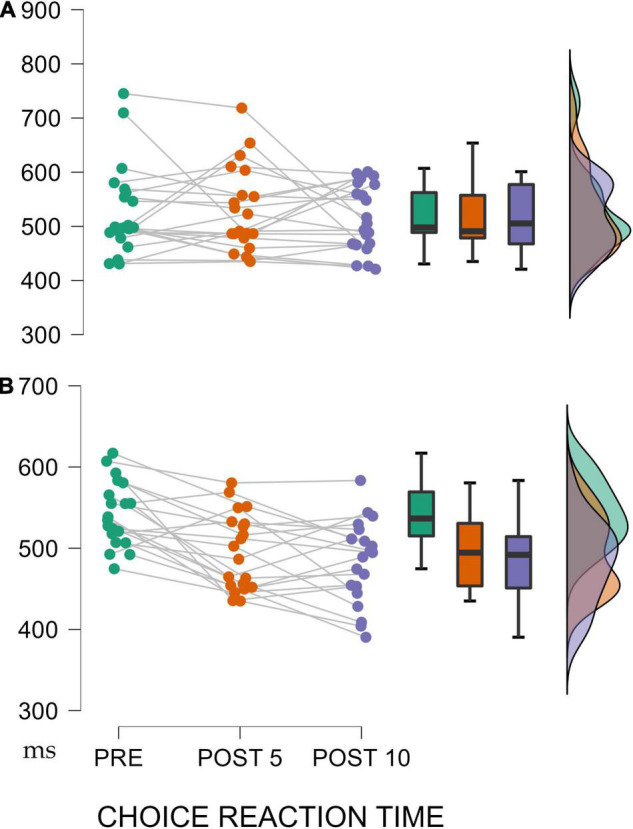
**(A)** Control Group. **(B)** Experimental Group. Evolution of CRT during the training process. At the beginning of the study (PRE), after 5 weeks of supervised training (POST 5) and after another 5 weeks of online training during quarantine (POST 10). Significant results shown with *p*-values are for the experimental group. Data are shown as mean ± standard error.

For the CRT, the TIME x GROUP showed a significant difference with large effect size [*F*_(1,40)_ = 3.602, *p* = 0.032, η_*p*_^2^ = 0.085]. The *post-hoc* Bonferroni analysis showed an improvement only in the experimental group during the intervention [PRE vs. POST5: *M* = 46.77, *SD* = 14.46, t(19) = 3.23, *p* = 0.027; PRE vs. POST10: *M* = 59.93, *SD* = 14.47, t(19) = 4.14, *p* = 0.001] (Figure 3).

**FIGURE 4:**
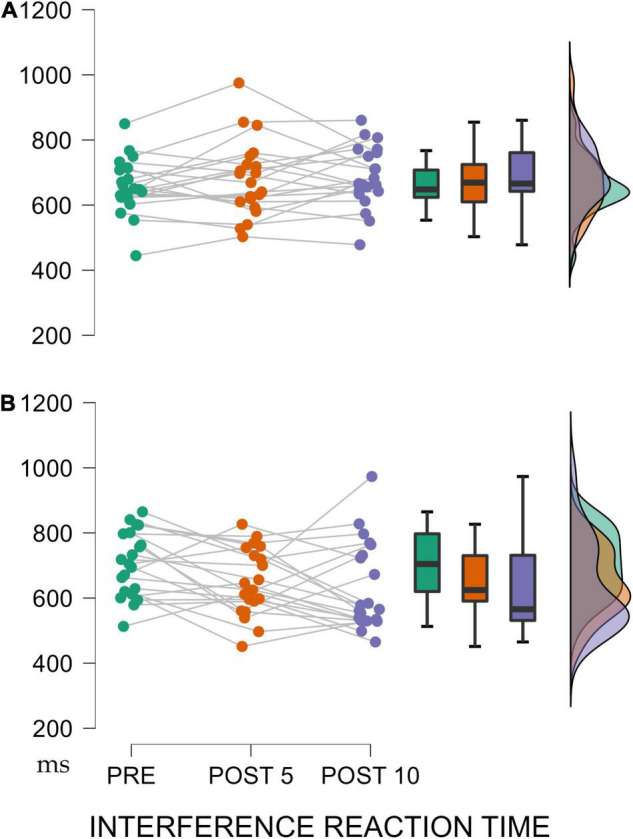
**(A)** Control Group. **(B)** Experimental Group. Evolution of InT during the training process. At the beginning of the study (PRE), after 5 weeks of supervised training (POST 5) and after another 5 weeks of online training during quarantine (POST 10). Significant results shown with *p*-values are for the experimental group. Data are shown as mean ± standard error.

For the InT, the TIME x GROUP showed a significant difference with a large effect size [*F*_(1,40)_ = 5.664, *p* = 0.005, η_*p*_^2^ = 0.124]. Furthermore, the *post-hoc* Bonferroni analysis showed an improvement only in the experimental group during the intervention and only at the end of the 10-week training program [PRE vs. POST10: *M* = 73.61, *SD* = 23.028, t(19) = 3.19, *p* = 0.030] (Figure 4).

**FIGURE 5:**
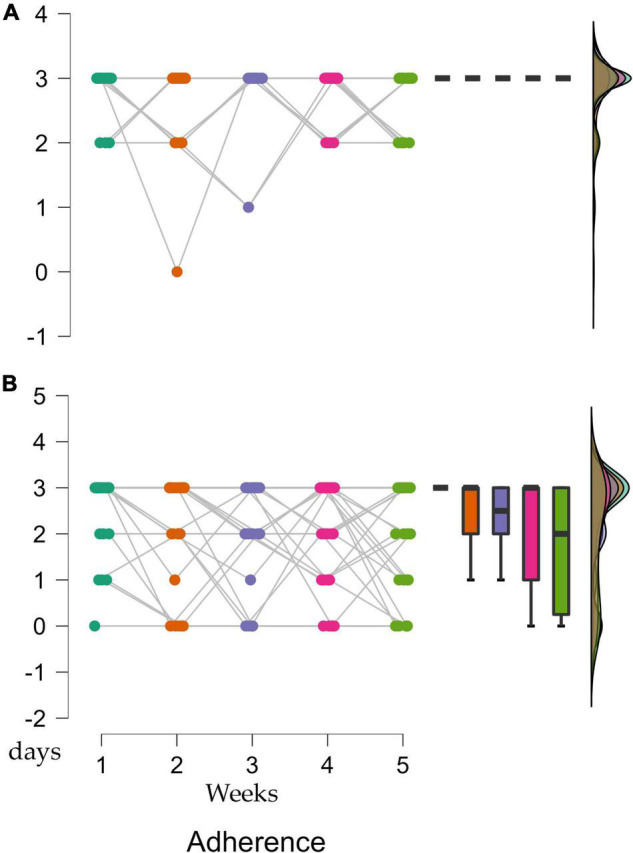
**(A)** Adherence during the supervised period. **(B)** Adherence during the online period. Weekly adherence in the supervised (weeks 1–5) and online (weeks 6–10) periods. Differences were only present during the online training period.

There were differences in adherence to the sessions between the two periods (supervised vs. online). There was 94% adherence during the supervised period and 71% adherence during the lockdown. An RM ANOVA was done to compare the evolution of the five sessions developed under supervision and online (RM ANOVA 5 × 2, a repeated measure for sessions, and between-subject factor for supervised vs. online training). When we compared the attendance of the participants to the sessions, there was a significant reduction in adherence to the online program [*F*_(1, 58)_ = 3.179; *p* = 0.014; η_*p*_^2^ = 0.052]. The Bonferroni *post-hoc* analysis showed a significant reduction in attendance to session 3 [*M* = 0.53, *SD* = 0.16, t(29) = 3.31, *p* = 0.049], 4 [*M* = 0.53, *SD* = 0.16, t(29) = 3.31, *p* = 0.049] and 5 [*M* = 0.83, *SD* = 0.16, t(29) = 5.17, *p* < 0.001] compared to the first online session (Figure 5).

Finally, the effects of training on wellbeing were analyzed, taking into account three variables (positive affect, negative affect, and subjective vitality). The analysis was done for the three temporal moments (PRE, POST 5, POST 10) and the two groups (experimental vs. control group). The repeated-measures ANOVA revealed the absence of significant differences for the three variables, positive affect [*F*_(1_, _40)_ = 2.34, *p* = 0.11, η_*p*_^2^ = 0.11], negative affect [*F*_(1_, _40)_ = 0.21, *p* = 0.979, η_*p*_^2^ = 0.001], and subjective vitality, [*F*_(1_, _40)_ = 0.88, *p* = 0.42, η_*p*_^2^ = 0.04]. Therefore, the results show that neither the supervised nor the online training phase affected wellbeing. Moreover, we saw a strong correlation between the three variables in the population (Table 2).

**TABLE 2:** Correlations between wellbeing variables.

	Variable	Positive affect	Negative affect	Vitality
**Pearson’s correlations in PRE**				
Negative affect	*n*	42	—	
	Pearson’s r	−0.578***	—	
	*p*-value	<0.001	—	
Vitality	*n*	42	42	—
	Pearson’s r	0.665***	−0.463**	—
	*p*-value	<0.001	0.002	—
**Pearson’s correlations at POST 5**				
Negative affect	*n*	41	—	
	Pearson’s r	−0.531***	—	
	*p*-value	<0.001	—	
Vitality	*n*	41	41	—
	Pearson’s r	0.797***	−0.551***	—
	*p*-value	<0.001	<0.001	—
**Pearson’s correlations at POST 10**				
Negative affect	*n*	42	—	
	Pearson’s r	−0.367*	—	
	*p*-value	0.017	—	
Vitality	*n*	42	42	—
	Pearson’s r	0.712***	−0.349*	—
	*p*-value	<0.001	0.024	—

*p < 0.05, **p < 0.01, and ***p < 0.001.

All the analyses were repeated considering the participant’s sex, and no differences were found in any variable in the TIME × GROUP × SEX analysis [CRT *F*_(3,37)_ = 0.431, *p* = 0.65, η_*p*_^2^ = 0.01; InT *F*_(3,37)_ = 0.274, *p* = 0.76, η_*p*_^2^ = 0.007; Positive Affect *F*_(3,37)_ = 1.54, *p* = 0.22, η_*p*_^2^ = 0.04; Negative Affect *F*_(3,37)_ = 1.58, *p* = 0.21, η_*p*_^2^ = 0.04; Vitality *F*_(3,37)_ = 0.783, *p* = 0.46, η_*p*_^2^ = 0.02].

## Discussion

The objective of this study was to determine whether HIFT could improve cognitive functioning, measured as complex RT, and wellbeing, measured as vitality and positive and negative affects, in young college students. Although the participants declared a sedentary lifestyle during the previous 6 months of the study, their VO_2_ max were within the normative values provided in the literature (Dwyer et al., 2005). As a result of the COVID-19 lockdown, a second objective, to analyze these variables and adherence during a quarantine period with online training, was included.

Regarding the participants’ processing speed, we observed that 5 weeks of HIFT supervised training improved their RT (CRT and InT). Our findings are in accordance with the literature, providing moderate to strong evidence that exercise being performed at moderate to vigorous exercise intensity can improve processing speed (Erickson et al., 2019).

The different neurophysiological mechanisms that underlie such functional changes might explain the benefits of chronic PE for processing speed. For example, angiogenesis and neurogenesis are structural brain changes mediated by exercise (van Praag et al., 2005). BDNF has been shown to be an essential neurotrophin in this process (Notaras and van den Buuse, 2019). In this sense, a previous study observed that a 3-month HIFT program significantly increased resting BDNF levels in young adults (Murawska-Cialowicz et al., 2015). The chronic changes in the brain must be differentiated from the acute effects. It seems that the benefits of exercise on complex RT may, as an acute response, be mediated by transient phenomena such as the release of catecholamines at the central level (Meeusen and De Meirleir, 1995), which modulate information processing (Robbins and Everitt, 1995).

However, we still do not entirely understand the mechanism related to cognitive improvement (Stillman et al., 2020). In this way, Stillman et al. (2016) proposed a multilevel system to study the underlying changes in cognitive functioning. Thus, cognitive changes can be produced by (a) molecular and cellular modifications (level 1), (b) structural and functional brain changes (level 2), and (c) behavioral and socioemotional changes (level 3). However, traditionally, the literature has focused on the first two levels, and there is a lack of evidence to understand the relations between behavioral and socioemotional changes with cognitive functioning (Stillman et al., 2016). Therefore, our study aimed to analyze the relationship between cognitive functioning and wellbeing, focusing on level 3 changes. Our hypothesis considered that hedonic and eudemonic variables of wellbeing would improve together with the RT variables.

However, the study results show that neither a 5-week HIFT supervised training program nor the online training during the COVID-19 quarantine affected this variable. Regarding the effect of training on these variables, the data contradict previous reports in the literature where acute, chronic PE and PT have been considered an effective tool to improve the wellbeing of young students (Cervelló et al., 2014; Garcia et al., 2015; Eather et al., 2019). However, some studies have found that high-intensity training did not cause significant changes in the wellbeing of this same population (Costigan et al., 2016). Moreover, data suggests an attenuated impact of the COVID-19 quarantine on the wellbeing variables in young students who presumably would not be burdened with responsibilities to the same degree as the adult population and thus experience less emotional distress (Brooks et al., 2020).

It has been shown that changes in cognitive functioning could be influenced by aspects of supervised training linked to mood and social relationships [could be related to level 3, as was proposed by Stillman et al. (2016)], such as (a) creating a structured and collaborative environment that favors positive reinforcement by peers and coaches; (b) a positive perception of social support; (c) achievement of goals; and (d) positive group dynamics (Lieberman et al., 2014). In agreement with these studies, our cognitive functioning improvements were higher during the supervised training than during the online training. However, improvements in processing speed were sustained through online training during the lockdown, unaffected by the reduction in adherence and social interactions. PE was probably sufficient to maintain unaltered mood and wellbeing, and the changes in processing speed could have been mediated by level 1 and 2 mechanisms (Stillman et al., 2016).

Although wellbeing was not modified due to the lockdown, significant differences in adherence were observed between the supervised and online phases. Self-determination theory (SDT) can explain the decrease in adherence (Deci and Ryan, 1985, 2000). SDT describes three psychological needs (autonomy, competence, and relatedness) that must be satisfied to increase the intrinsic motivation for a given activity. In our online training program, some of these needs were not satisfied, such as relatedness (Deci and Ryan, 1985) with peers and coaches, which has a significant impact on adherence to exercise (Kang et al., 2019), or the supervision of qualified professionals (Garber et al., 2011). In addition, recent studies have shown that people who reduced their PA during lockdown experienced decreased wellbeing (Brand et al., 2020; Faulkner et al., 2021). However, in our results, despite the correlation between positive and negative affect with vitality, the latter was not reduced in parallel with a decrease in adherence. Moreover, CRF is related to vitality in young people (Eddolls et al., 2018), and CRF can be maintained with a lower volume of exercise if the intensity of the activity is conserved (Spiering et al., 2021). Given that there were no changes in the exercise intensity throughout the training program, the reduction in adherence in this study probably did not affect CRF, and hence, vitality was not altered.

Our study demonstrates that HIFT can improve cognitive functioning in young university students and could be successfully applied through online training under lockdown circumstances, maintaining the exercise intensity and improving cognitive functioning. Moreover, as we have demonstrated, it is possible to maintain the subjective exercise intensity (measured by RPE) of the HIFT training in the online approach, with no adverse changes in wellbeing. However, if we want to use this methodology in future lockdowns, it would be interesting to analyze the lockdown training protocols from the SDT perspective, trying to overcome psychological needs deficiencies during the quarantine periods.

Finally, our study did not find any sex differences in the analyses performed. A previous meta-analysis showed discrepancies in sex differences. For example, Barha et al. (2017) found that sex was a strong moderator in the exercise-cognition relationship in their meta-analysis, with more pronounced improvements for women. However, Falck et al. (2019) did not find this relationship in their meta-analysis. In any case, both meta-analyses were done in older people. Some old studies showed that older women participated in lower leisure-time PA than older men (Lee, 2005), or reported lower frequencies of physical activities (Kaplan et al., 2001). In addition, sedentarism has a worse cognitive impact in women than in men (Fagot et al., 2019). Thus, Barha and Liu-Ambrose (2018) proposed that the increase in daily PA could be greater for older women than for older men, and this difference could promote more significant cognitive improvements in women. However, recent studies have shown that, nowadays, older women are less sedentary and do more PA than men (Wennman et al., 2019; Lopez-Valenciano et al., 2020). Suppose PA determines the cognitive functioning improvements after a PT period. In that case, present and future research will find a sex gap with better improvements in older men.

Regarding this study, we do not know any review of sex differences in young university students on the exercise-cognition relationship. Thus, according to the results of our study, sex does not moderate the improvement in processing speed after chronic exercise in young people.

This study investigated whether, in young adults, supervised and unsupervised HIFT can influence wellbeing and cognitive functioning, as empirical evidence regarding the effect of PEs on wellbeing and cognitive functioning is scant in this age group (Stillman et al., 2020). However, we did not find evidence that exercise-related changes in socioemotional parameters are associated with alteration of cognitive functioning, as previously proposed (Stillman et al., 2016). We described our training methodology with HR records and used ventilatory thresholds to control the exercise load. The implications that arise from the findings of our study broaden our knowledge on the influence of HIFT on cognitive functioning and wellbeing in younger adults.

## Limitations

As a result of lockdown, the main limitation to this study was that it was impossible to carry out post-training fitness tests, so we do not know if the observed changes in cognitive functioning are related to improvements in fitness. Therefore, it would be interesting for future research to look into the implicit mechanisms related to physical fitness that could potentially explain improvements in complex RT. Moreover, the study did not employ allocation concealment, which indicates a possible source of bias.

## Conclusion

On the one hand, HIFT training improves processing speed, measured through two complex RT tasks (CRT and InT) in young college students, without mediation by changes in psychological wellbeing. Also, it was observed that a training program during a quarantine period, forced by the global COVID-19 pandemic, promoted the maintenance of RT improvements previously achieved during a supervised training program. On the other hand, it was observed that this situation did not have adverse effects on this population’s wellbeing. However, it negatively affected adherence to the online training program, possibly because of the suppression of typical factors linked to training supervision and interactions with peers and coaches.

## Data Availability Statement

The raw data supporting the conclusions of this article will be made available by the authors, without undue reservation.

## Ethics Statement

The studies involving human participants were reviewed and approved by the Miguel Hernández University Ethics Committee (UMH.CID.DPC.02.17). The patients/participants provided their written informed consent to participate in this study.

## Author Contributions

JB-F, LC-H, DP, and EC contributed to the conception and design of the study. JB-F organized the database. JB-F, LC-H, and DP performed the statistical analysis and wrote the final manuscript. JB-F and LC-H wrote the first draft of the manuscript. All authors contributed to manuscript revision, read, and approved the submitted version.

## Conflict of Interest

The authors declare that the research was conducted in the absence of any commercial or financial relationships that could be construed as a potential conflict of interest.

## Publisher’s Note

All claims expressed in this article are solely those of the authors and do not necessarily represent those of their affiliated organizations, or those of the publisher, the editors and the reviewers. Any product that may be evaluated in this article, or claim that may be made by its manufacturer, is not guaranteed or endorsed by the publisher.

## Footnotes

1
http://www.gpower.hhu.de/
